# Emerging Vapor-Phase Growth Methods for Halide Perovskite Thin Films and Nanostructures

**DOI:** 10.1021/acsmaterialsau.5c00122

**Published:** 2025-12-11

**Authors:** Pushpender Yadav, Ajay Sah, Caitlin N. Ewald, Seokhyoung Kim

**Affiliations:** Department of Chemistry, 3078 Michigan State University, East Lansing, Michigan 48824, United States

**Keywords:** vapor deposition, physical vapor deposition, chemical vapor deposition, metal halide perovskite, thin films, nanowires, nanoplatelets, micropatterns

## Abstract

Metal halide perovskites (MHPs) have emerged as a class of highly efficient semiconductor materials, attracting widespread interest for their potential applications in photovoltaics, light-emitting diodes, photodetectors, and transistors. Vapor-phase techniques for perovskite synthesis represent a paradigm shift in materials processing, offering precise control over morphology, crystallinity, and composition, while enabling industry-scale manufacturing. This review highlights advancements in vapor-phase synthesis of MHP thin films and micro- and nanostructures, emphasizing strategies to enhance electronic and optical properties, with structural control for next-generation device integration. We introducethe fundamental principles of widely used vapor-phase growth methods and discuss key process modifications employed to control chemical composition, crystal dimension, anisotropic growth, doping, phase purity, and substrate compatibility. We also summarize notable device performance, underscoring the transformative potential of vapor-phase approaches for scalable and high-performance optoelectronic technologies with MHPs.

## Graphical abstract

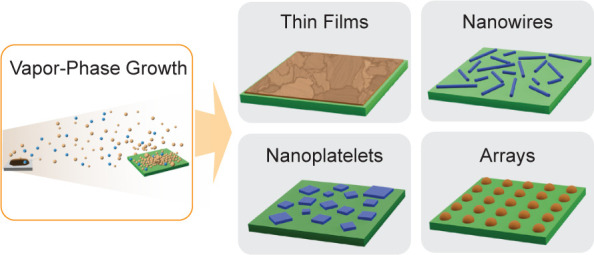

## 1 Introduction

Metal halide perovskites (MHPs) have emerged as a promising class of semiconducting materials owing to their exceptional optoelectronic properties, including direct and tunable bandgaps, high absorption coefficients, long carrier diffusion lengths, and remarkable defect tolerance.^1–4^ These properties have positioned MHPs as leading candidates for photovoltaic and light emitting applications. Notably, MHP thin film solar cells have now achieved power conversion efficiencies (PCEs) exceeding 26% in single-junction configurations,^5–7^ a performance often attributed to the intrinsic defect tolerance, which enables high efficiency across a wide range of fabrication methods. Unlike conventional semiconductors that demand high crystallinity and minimal defect densities, MHPs can accommodate relatively high concentrations of intrinsic defects with minimal impact on their optoelectronic performance.^8–10^

Numerous synthetic strategies have been developed for MHPs, with solution-phase methods receiving significant attention due to their low-cost processing, and low-temperature reaction conditions enabling the synthesis of thin films and nanostructures with scalable, large-area device fabrication.^11–14^ High-performance MHP films have been prepared via one-step and two-step spin coating, solution evaporation, and inkjet printing, yielding high PCEs with tunable film quality.^15–19^ Additionally, solution-based methods have been adapted to synthesize nanostructures such as colloidal quantum dots (QDs), nanowires (NWs), and nanoplatelets (NPLs) with control over morphology, size distribution, and surface chemistry. Techniques such as hot-injection, ligand-assisted reprecipitation (LARP), and solvothermal crystallization have proven effective for generating highly luminescent nanocrystals. These nanostructures exhibit low lasing thresholds and strong photodetection responses, making them attractive for emerging optoelectronic, photonic, and sensing applications.^1,3,20,21^

Despite these advantages, solution-based techniques face persistent challenges including uncontrolled nucleation and growth, solvent impurities, phase inhomogeneity, and suboptimal interface quality, all of which contribute to variability in device performance and long-term operational stability. Moreover, complex heterostructures such as multiquantum wells (MQWs) and epitaxial superlattices with atomically sharp interfaces remain difficult to access using solution-based approaches alone.

Vapor-phase growth techniques offer a compelling approach to overcome these limitations, providing solvent-free, highly controllable environments for the growth of MHP thin films and nanostructures. Methods such as physical vapor deposition (PVD), chemical vapor deposition (CVD), and metal–organic chemical vapor deposition (MOCVD) enable precise control of growth kinetics, film composition, morphology, and interface quality. These attributes are critical for achieving high structural order, uniformity, and well-defined interfaces. Vapor-phase methods have proven particularly effective in producing high-quality films and nanostructures via atomically controlled epitaxial growth for high-performance devices. For instance, high-quality III–V compound semiconductor (*e.g*., GaAs or GaN) thin films, vital for advanced optoelectronic applications, are widely produced using vapor-phase deposition techniques with ultrahigh purity and precise structural control.^22^ Additionally, catalyzed growth mechanisms such as vapor–liquid–solid (VLS) growth have enabled the controlled synthesis of crystalline NWs with tunable diameters, orientations, and doping levels.^23–25^ Large-area CVD growth of MoS_2_ monolayers on SiO_2_ substrates have demonstrated the scalability, crystallinity, and electronic performance of a vapor-grown two-dimensional (2D) transition metal dichalcogenide (TMD).^26^ Furthermore, vapor-phase deposition allows for precise control of grain boundary structures and the formation of large, continuous monolayer domains.^27^

In this review, we present a focused overview of recent progress in the vapor-phase synthesis of MHPs thin films, micro and nanostructures.^28–35^ We begin by outlining the commonly employed vapor-phase methods along with their underlying working principles. Building on these methodologies, we then discuss key advancements and modifications to these techniques that result in controlled growth of MHP thin films, nanostructures including 1D NWs, 2D NPLs, and periodic arrays of these structures, highlighting notable device performances. Finally, we conclude with possible future vapor phase methodologies that can open new avenues for MHPs synthesis.

## 2 Vapor-Phase Growth Methods

In this section, we categorize vapor-phase growth techniques into four distinct types based on their operational complexity and chemical involvement. We begin with PVD (section 2.1), which involves the direct physical transfer of material with no or minimal chemical reactions. We then introduce CVD (section 2.2) and MOCVD (section 2.3), where precursors undergo chemical transformation at the surface of substrate. Finally, we discuss sequential or multistep methods (section 2.4), which combine aspects of physical and chemical processes through controlled, stepwise deposition to achieve enhanced structural and compositional control. This categorization provides a progressive understanding of vapor-phase synthesis strategies and has been summarized in Table 1.

**Table 1:** Summary of Vapor-Phase Methods

Method	Description	Strengths	Limitations	Refs
PVD	Sublimation or evaporation of precursors; sequential or coevaporation of organic and inorganic sources	Precise flux and thickness control; smooth, compact films with high reproducibility; fewer conversion steps	Stoichiometry drift with volatile halides; residual PbX_2_ phases; requires *in-situ* calibration for scaling uniformity	^31,37,45–49,52,53,56,57^
CVD	Reactants transported in carrier gas; reaction on heated substrate	Good coverage on substrates; parametrized mass transport; scalable reactors; adjustable *via* precursor flow, temperature, and pressure	Gas-phase side reactions before/during deposition; precursor decomposition or volatility mismatch; narrow temperature range for stable growth; nonuniform film thickness from uneven vapor flow; reproducibility and scale-up difficulties	^24,33–35,58,60,66,67^
MOCVD	Metal–organic, halide, and amine precursors delivered into reactor for surface reaction	Potential for epitaxial and orientation-controlled films; precise layer-by-layer thickness control	Limited precursor availability/reactivity for MHPs; complex gas handling and ligand chemistry	^28,71–75^
Hybrid/sequential	Stepwise chemical conversion of predeposited precursors (*e.g*., PbI_2_) to desired MHP thin films; sequential vapor exposure for halide exchange	Enables compositional tuning (*e.g*., halide exchange); retains film morphology; pinhole-free films	Residual PbX_2_ or secondary phases from limited diffusion; conversion mainly along grain boundaries	^85–92^

### 2.1 Physical Vapor Deposition (PVD)

PVD encompasses a suite of well-established vacuum-based thin-film deposition techniques that rely on physical processes to vaporize target materials and condense them onto substrates as solid films. These techniques typically employ solid precursors that are vaporized into the gas phase and transported through a vacuum environment to a substrate, which is often maintained at or near ambient temperature. Upon reaching the substrate surface, the vapor condenses to form a uniform thin film as shown in Figure 1A. Common methods of material vaporization include thermal evaporation, electron beam (e-beam) evaporation, pulsed laser deposition (PLD), and sputtering. These techniques may utilize single-source configurations or multisource systems in which each target material is independently controlled and monitored, enabling precise stoichiometric tuning and improved film uniformity. Thermal evaporation employs resistive heating to vaporize a target. This method is effective for materials with low to moderate melting points. This has been largely demonstrated with hybrid organo-lead MHPs,^36,37^ where crystal grain size can be influenced by controlling substrate temperature.^38–40^ Recent demonstrations of thermally evaporated devices have surpassed 24% PCE, showing that carefully optimized vacuum-grown films can compete directly with the best solution-processed counterparts.^41^ Importantly, one of the most promising applications of PVD-deposited MHP is in tandem architecture, where bandgap tuning and precise thickness control are essential. For example, four-source vacuum deposition to fabricate wide-bandgap FA_0.7_Cs_0.3_Pb­(I,Br)_3_ (FA: formamidinium) layers for all-perovskite tandems, achieving open-circuit voltages above 2.0 V and efficiencies exceeding 24% in two-terminal tandem devices.^42^ E-beam evaporation is commonly employed for high-melting point materials. A focused high-energy electron beam selectively heats a localized region of the target material, allowing vaporization without raising the temperature of the entire volume.^43^ While the use of e-beam evaporation in MHP synthesis is still relatively limited, it has been employed to fabricate CsPbBr_3_ thin films.^44^ PLD involves the ablation of the target material using short, high-intensity laser pulses, commonly in the ultraviolet range. This method has been successfully employed for materials such as CsPbX_3_ (X = Cl, Br, I),^45,46^ MAPbX_3_ (MA: methylammonium),^47–49^ CsSnX_3_,^50^ double perovskites (DP) such as Cs_2_AgBiBr_6_,^51^ among others.^52,53^ Sputtering is an evaporation method that works by bombarding the material with high energy ions from plasma, where atoms are ejected from the surface of the target material. Sputtering has been successfully employed to synthesize thin films of CsPbBr_3_ and MAPbI_3_ using single-target sources.^54,55^

**Figure 1:**
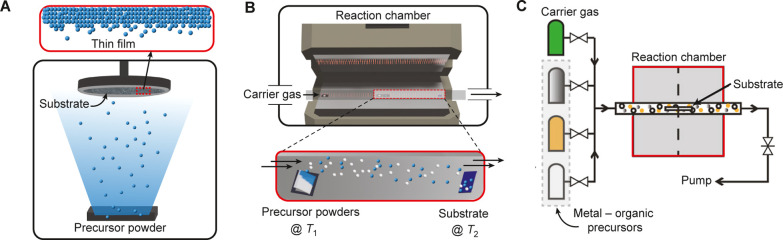
Distinct vapor-phase growth methods. (A) PVD. (B) CVD. (C) MOCVD, reproduced from ref.^28^ Copyright 2025 American Chemical Society.

These PVD techniques provide precise control over the delivery of inorganic precursors; however, maintaining accurate stoichiometry in hybrid perovskite films requires careful management of volatile organic components such as MAI or FAI. Because these species exhibit high vapor pressures, their incorporation efficiency strongly depends on substrate temperature and chamber pressure, where a narrow operational window determines whether films remain stoichiometric or become A-site deficient.^56^ Studies have shown that balancing precursor flux and desorption kinetics is critical for reliable composition control and reproducible film quality. Ensuring process reproducibility further requires accurate calibration of deposition rates and *in situ* thickness monitoring, as small deviations in flux or temperature can cause large variations in film thickness and morphology. In practice, reproducibility and uniformity improve when the relationships among precursor ratio, substrate temperature, and background pressure are systematically mapped and optimized. Moreover, PVD enables advanced film architectures such as graded compositions and homojunctions, where controlled compositional gradients across the film thickness induce internal electric fields that enhance carrier transport and device performance.^57^ Together, these considerations represent key advantages of PVD that enable the growth of reproducible, tunable, and high-quality MHP thin films.

### 2.2 Chemical Vapor Deposition (CVD)

CVD is fundamentally a nonequilibrium, kinetically driven process^58^ where inorganic or organic volatile precursor are transported to a substrate surface held at a high temperature and undergo a series of physical and chemical transformations, ultimately resulting in the deposition of a solid product. While traditional CVD processes often begin directly with volatile precursors, for MHPs, deposition frequently initiates from solid precursors that are thermally vaporized. As depicted in Figure 1B, an inert carrier gas, typically argon (Ar), hydrogen (H_2_), or nitrogen (N_2_), is employed to transport the vapor-phase reactants to a substrate maintained at an elevated temperature. Upon reaching the substrate, the precursors deposit on the substrate, forming solid perovskite structures. The nonequilibrium nature of CVD allows kinetic control over the reaction, enabling the formation of metastable phases,^59^ precise morphology,^35,60^ tailored crystallinity,^61^ exceptionally high doping level,^62^ and controlled stoichiometry^63,64^ that are often not achievable under equilibrium conditions. CVD growth is influenced by several key parameters, including the precursor ratio, substrate temperature, material composition, ambient pressure, carrier gas flow rate, reaction duration, and cooling conditions. The location of the substrate within the reaction chamber is also a consideration. For example, in a tube furnace system, where the precursors and substrate are placed in a quartz tube that is heated by one^33,65^ or multiple zones,^66,67^ the distance of the substrate from the precursors impacts the result of the deposition. CVD also facilitates direct epitaxial^68^ or aligned^69^ crystal growth through the use of specific substrates such as mica or sapphire, whose anisotropic surface features guide the directional growth of nanostructures. Moreover, the combination of lithography and CVD to synthesize perovskite arrays has enabled the fabrication of integrated perovskite devices suitable for a range of optoelectronic applications.^70^ VLS growth has also been achieved for MHP NWs^24,34^ through the use of a metal nanoparticle catalyst, which facilitates precursor decomposition and material incorporation at the liquid droplet-solid material interface.

### 2.3 Metal–Organic Chemical Vapor Deposition (MOCVD)

MOCVD is a subtype of CVD that employs volatile organo-metallic precursors. For instance, in MOCVD of III–V compound semiconductors, organometallic precursors such as trimethylgallium (TMGa) are delivered into the reaction chamber by carrier gas along with reactive group-V precursor gases like arsine (AsH_3_) or phosphine (PH_3_). These precursors undergo thermal decomposition or react near the substrate, enabling atomic-level control over thin films as shown in Figure 1C, with volatile byproducts efficiently removed. MOCVD operates at lower growth temperatures than traditional CVD, which is advantageous for preventing thermal damage and enabling the integration of temperature-sensitive substrates. MOCVD also enables the growth of multicomponent materials with excellent compositional uniformity and conformality, even with complex geometries.^71–75^ While significant progress has been made in MOCVD for the synthesis of III–V (GaN, InP, and InGaAs), and oxide semiconductors,^76–81^ its application to halide perovskites remains in its early stages. A recent notable advancement includes the successful MOCVD growth of MAPbX_3_ thin films.^28^

### 2.4 Hybrid and Sequential Methods

Hybrid deposition strategies that integrate solution deposition, PVD, and CVD techniques have emerged as effective routes for synthesizing high-quality MHP thin films and nanostructures. In a common approach, a metal halide layer is first deposited via thermal evaporation or spin coating, followed by a PVD or CVD step in which an organic halide, such as MAI, is introduced to form the perovskite structure.^82,83^ This approach has been further adapted to enable compositional tuning, wherein a perovskite film deposited by thermal evaporation undergoes subsequent CVD processing to incorporate alternate cations or halides,^84^ resulting in a mixed-cation or mixed-halide perovskite. Hybrid or sequential vapor-phase approaches have enabled not only better control over crystallization dynamics but also offered unique opportunities to investigate the evolution of film growth in sequential stages. The exploration of multistep processes, substrate orientation effects, vapor transport mechanisms, and recrystallization phenomena has revealed new pathways for enhancing film quality and device performance.^85–92^

## 3 MHP Thin Films

In this section, we summarize advances in the vapor-phase synthesis of MHPs thin films grown *via* PVD (section 3.1) (Table 2), CVD (section 3.2) (Table 3), MOCVD (section 3.3), and sequential chemical conversion (section 3.4) (Table 4).

**Table 2:** Summary of PVD-Grown Thin Films

Material	Key Observations	Properties	Refs
MAPbI_3_	Smooth, compact films, uniform coverage	Device-quality perovskites; reduced pinholes; scalable deposition	^31^
MAPbI_3_	Surface topography shows reduced roughness	Optimized morphology improves charge transport and uniformity	^93^
MAPbI_3‑x_Cl_*x*_	Vapor →dense and uniform; solution → larger grains, more pinholes	Vapor → better control; solution → cheaper, less uniform	^37^
2D/3D heterojunctions	Layered/bulk hybrid structures	Improves stability and exciton confinement in LEDs/solar cells	^32^
MAPbI_3_	Controlled stoichiometry *via* substrate temperature tuning	20.6% stabilized PCE	^56^
Cs_2_AgBiBr_6_	Low *P* → porous; high *P* → denser, smoother	Pressure tuning enables morphology control and phase purity	^51^

**Table 3:** Summary of CVD-Grown Thin Films

Material	Key Observations	Properties	Refs
MAPbI_3‑x_Cl_*x*_	Dense coverage, controlled morphology	High-quality films with uniform stoichiometry; reproducible coverage; improved grain size	^66^
MAPbI_3_	PbAc_2_ → smoother, larger grains; PbI_2_ → incomplete conversion	Precursor selection strongly affects crystallinity, stoichiometry, and defect density	^94^
Cs_2_SnI_2_Cl_2_/Cs_2_PbI_2_Cl_2_	Large grains, 2D RP structure verified by XRD & PL	Lead-free perovskites; high crystal quality; good excitonic features	^95^
Cs_3_Bi_2_Br_9_	CsBr:BiBr_3_ molar ratio; 1:1 → rough and porous; 1.5:1 → compact and smooth; 3:1 → phase impurity	Molar ratio (CsBr:BiBr_3_) controls morphology and electronic quality	^67^
CsPbBr_3_/CsSnBr_3_	Vapor-phase epitaxy on mica and sapphire yields oriented, crystalline films	Improved carrier transport and stability; benchmark for scalable perovskite epitaxy	^96–98^

**Table 4:** Summary of Thin Films Grown by Hybrid and Sequential Chemical Conversion

Material	Key Observations	Properties	Refs
CsSnI_3_:PbI_2_	Sequential evaporation followed by postannealing yields compact polycrystalline films	Demonstrates solid-state interdiffusion between precursors; tunable stoichiometry and dense coverage	^85,100^
BA/MA perovskites	100% BAI → 2D; 0% BAI → 3D	Dimensionality tuning alters bandgap, stability, and performance	^99^
MAPbI_3_	Uniform pinhole-free films obtained by dual-zone VTD	Scalable process producing smooth morphology and uniform thickness; compatible with large-area coating	^86^
MAPbBr_3_	Separate PbBr_2_ and MABr sources react in gas phase, forming compact MAPbBr_3_ layers	Efficient halide incorporation and high conversion rate; improved crystallinity	^87^
MAPbI_3_	Postannealing reduces roughness and enhances grain connectivity	Enhanced charge transport and reduced trap density relative to one-step growth	^88^
MAPbI_3_	Temperature gradient (*T*_2_:160 °C/*T*_1_:190 °C) optimizes uniformity and phase formation	Defines process window for reproducible morphology; balances kinetics vs diffusion	^89^
MAPbI_3_	MAI vapor converts predeposited PbI_2_ film into perovskite without residual PbI_2_	Yields dense, stoichiometric films with excellent optical quality	^90^
MAPbI_3_	Liquefaction and recrystallization by amine vapor treatment improves crystallinity	Grain size growth from ∼300 nm to >400 μm	^91^
MAPb(I_1‑*x*_Br_*x*_)_3_	Sequential halide vapor treatments create surface-composition gradients and color change	Enables tunable bandgap and enhanced stability; demonstrates controllable halide exchange	^92^

### 3.1 PVD-Grown Thin Films

A widely adopted approach for the fabrication of organic–inorganic hybrid perovskite thin films is single-source PVD (SSPVD), which utilizes the direct sublimation of presynthesized perovskite powder.^31^ As illustrated in Figure 2A (left), the perovskite source material is rapidly heated, enabling the powder to transition directly to the vapor phase. The vapor is subsequently transported to the substrate, where it condenses into a uniform thin film. This method has been utilized to yield MAPbI_3_ films with uniform surface coverage, and well-defined grain structures (Figure 2A, right), achieving a PCE of 10.9% with improved moisture stability. Single-source flash evaporation (SSFE), another single-source method, utilizes rapid thermal vaporization of presynthesized perovskite powder, facilitating complete and uniform deposition.^93^ The fast heating rate of SSFE ensures homogeneous film deposition while preserving the precursor stoichiometry. The resulting ultrasmooth MAPbI_3_ films showed roughness of 5.23 nm, with localized regions reaching 2.86 nm (Figure 2B). Devices based on these films show exceptional photodetector performance, including a responsivity of 51 mA/W and a detectivity of 9.55 × 10^10^ Jones.

**Figure 2:**
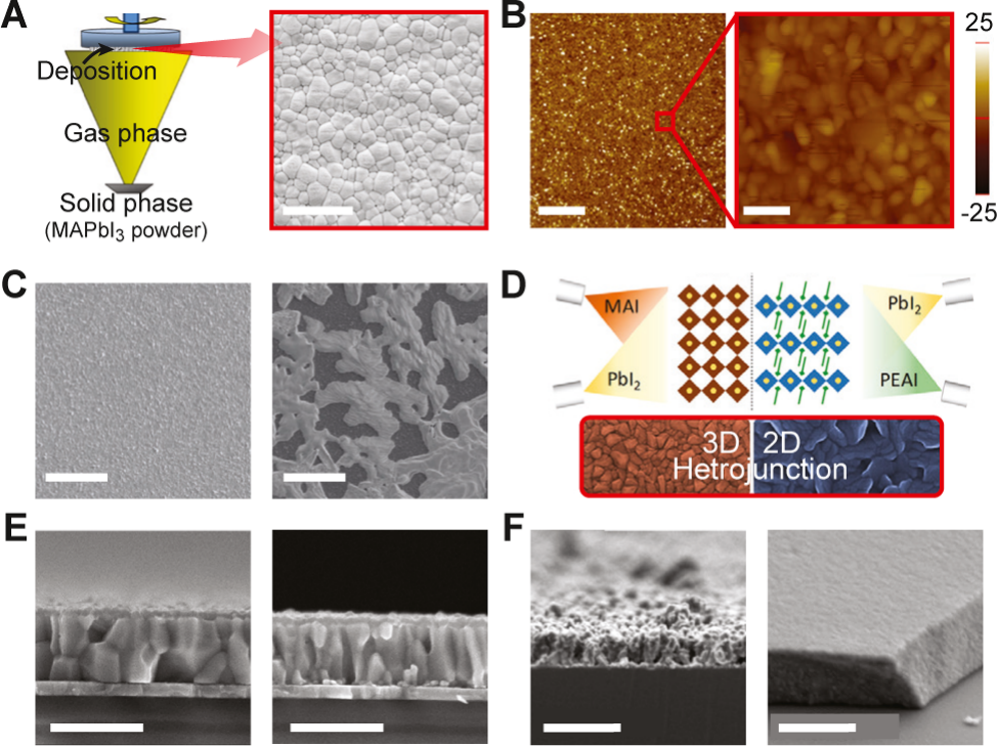
PVD-processed thin films. (A) PVD schematic (left) with SEM image of MAPbI_3_ thin film (right); scale bar: 5 μm. (B) AFM images of MAPbI_3_ film surface; scale bar: 5 μm (left), 200 nm (right). (C) SEM images of vapor-deposited (left) and solution-processed (right) MAPbI_3–*x*_Cl_*x*_ films; scale bars: 10 μm. (D) Schematic representation of 2*D*/3D heterojunction. (E) Films prepared on PTAA (left) and MeO-2PACz (right); scale bars: 800 nm. (F) Cs_2_AgBiBr_6_ thin films grown at lower (left) and higher (right) pressures; scale bars: 400 nm. (A) Reprinted with permission under a Creative Commons CC-BY 4.0 from ref. 31 Copyright 2016 Spring Nature. (B) Reprinted with permission under a Creative Commons CC-BY 4.0 from ref. 93 Copyright 2016 Spring Nature. (C) Reproduced with permission from ref. 37 Copyright 2013 Springer Nature. (D) Reproduced from ref. 32 Copyright 2019 American Chemical Society. (E) Reproduced from ref. 56 Copyright 2020 American Chemical Society. (F) Reproduced from ref. 51 Copyright 2021 American Chemical Society.

Multisource PVD (MSPVD) involves the separate yet simultaneous vaporization of solid sources and has demonstrated improved compositional control in thin films.^37^ Dual-source PVD (DSPVD) maintains a precisely controlled and sufficiently high precursor flux from both sources, ensuring a balanced and continuous supply of inorganic and organic species in well-defined proportions. This results in pure-phase MAPbI_3 – *x*_Cl_*x*_ films with minimal residues, mitigating the formation of undesirable secondary phases. Comparative SEM images (Figure 2C) highlight the superior film continuity and density, in contrast to the incomplete surface coverage of solution-processed films. Devices based on these films, configured in a planar heterojunction architecture, exhibit PCEs exceeding 15% with an open-circuit voltage (*V*_OC_) of 1.07 V. The DSPVD is further extended to the development of vacuum-deposited 2*D*/3D/2D perovskite heterojunctions (Figure 2D), which aims to achieve higher *V*_OC_ and enhanced stability.^32^ This approach creates well-defined interfaces between dimensional phases as shown in Figure 2D, illustrating the heterojunctions of the 3D MAPbI_3_ layer and the adjacent 2D perovskite layers. This study also demonstrated the potential for interface passivation, suppressing domain intermixing commonly observed in solution-processed 2D/3D systems. Another study using DSPVD demonstrated MAPbI_3_ thin film growth, revealing that substrate temperature governs both MAI incorporation and film stoichiometry.^56^ Near-room-temperature growth produced compact, stoichiometric MAPbI_3_ layers, while elevated temperatures led to PbI_2_-rich compositions. Cross-sectional SEM (Figure 2E) shows dense, columnar morphologies whose orientation and grain dimensions strongly depended on the underlying hole-transport layer, poly­[bis­(4-phenyl)­(2,4,6-trimethylphenyl)­amine] (PTA) versus [2-(3,6-dimethoxy-9H-carbazol-9-yl)­ethyl]­phosphonic acid (MeO-2PACz), indicating substrate-specific nucleation pathways. Under optimized conditions on MeO-2PACz, a stabilized PCE of 20.6% was achieved, representing the first *p–i–n* evaporated perovskite device exceeding 20%.

PLD represents another approach to grow perovskite thin films by using a high-energy pulsed laser to ablate a target material, generating a plasma plume that deposits onto the substrate. The integration of mechanochemical synthesis with PLD demonstrated an effective strategy for fabricating Cs_2_AgBiBr_6_ DP films.^51^ In this, mechanochemically presynthesized DP powder serves as the source material for PLD, with deposition carried out at a substrate temperature of 200 °C . The high-energy laser pulses facilitate the ablation of the target material with minimal compositional deviation, preserving the stoichiometry and phase of the original material. This results in the deposition of highly crystalline films with grain sizes exceeding 200 nm, making them suitable for UV and X-ray photodetection applications. Furthermore, the PLD process operates effectively across a range of pressures, as illustrated in Figure 2F.

### 3.2 CVD-Grown Thin Films

Early studies on the CVD synthesis of MHP thin films demonstrated the feasibility of *in situ* reaction of PbX_2_ and MAI vapors to form triiodide and mixed halide perovskite.^66^ Solar cells based on these thin films achieve a PCE of 11.1%. As depicted schematically in Figure 3A, this process involves the reaction of PbI_2_ or PbCl_2_ and CH_3_NH_3_I vapors in a tube furnace configuration, producing films with continuous coverage, well-defined micrometer-scale grains, and an average thickness of approximately 500 nm. Subsequent studies have underscored the critical role of precursor selection on film quality and device performance metrics. Comparative investigations employing either PbI_2_ or lead diacetate trihydrate (Pb­(CH_3_COO)_2_·3H_2_O, abbreviated as PbAc_2_) in combination with MAI were conducted in a two-zone quartz tube furnace under Ar flow.^94^ In the case of PbI_2_ and MAI, PbI_2_ was placed in the high-temperature zone and MAI in the cooler region, resulting in perovskite films that exhibited incomplete conversion but rather a metastable dark phase containing excess iodine and unreacted PbI_2_, as shown in Figure 3B (right). In contrast, employing PbAc_2_ as the lead precursor and placing MAI in the high-temperature zone yields a highly uniform MAPbI_3_ film with smoother morphology and larger grain sizes (∼0.5 μm), as shown in Figure 3B (left). Although devices fabricated using these films initially exhibited low efficiency (∼0.23% PCE), their performance improved to ∼5.5% following light soaking and dark storage.

**Figure 3:**
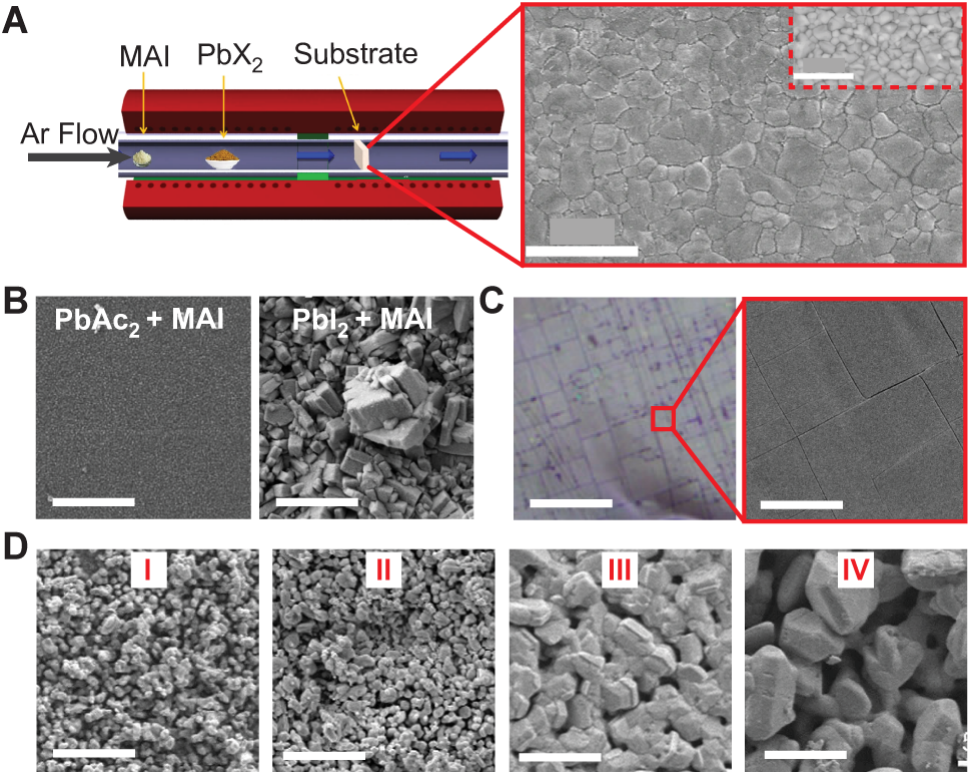
CVD-processed thin films. (A) CVD schematic (left) and SEM image (right) of a MAPbI_3–*x*_Cl_*x*_ film, inset displaying a backscattered electron (BSE) image; scale bars: 2 μm. (B) Films obtained from PbAc_2_ (left) and PbI_2_ (right); scale bars: 50 μm. (C) Cs_2_SnI_2_Cl_2_ with optical (left); scale bar: 100 μm, and SEM (right) image; scale bar: 20 μm. (D) SEM images of CBB films with molar ratios of CsBr: BiBr_3_, 0.67:1­(I), 1:1­(II), 1.5:1­(III), and 2:1­(IV); scale bars: 5 μm. (A) Reprinted with permission under a Creative Commons CC-BY 4.0 from ref. 66 Copyright 2015 Spring Nature. (B) Reproduced with the permission from ref. 94 Copyright 2024 John Wiley and Sons. (C) Reproduced from ref. 95 Copyright 2024 American Chemical Society. (D) Reproduced with the permission from ref. 67 Copyright 2024 Springer Nature.

Additionally, CVD has enabled the scalable synthesis of all-inorganic 2D RP perovskites. Centimeter-scale films of Cs_2_PbI_2_Cl_2_ and Cs_2_SnI_2_Cl_2_ (*n* = 1 phase) were successfully grown using a two-zone tube furnace, where CsI and CsCl and either PbI_2_ or SnI_2_ were evaporated under an inert Ar atmosphere.^95^ The resulting Cs_2_PbI_2_Cl_2_ films featured millimeter-scale lateral domains, with the Ruddlesden-Popper (RP) phase confirmed by sharp XRD peaks, as well as strong photoluminescence (PL) and excitonic absorption showcasing the 2D perovskite lattice structure. Cs_2_SnI_2_Cl_2_ films showed similar morphology and phase purity, as shown in Figure 3C. Another notable advancement is the development of high-quality vacancy-ordered Cs_3_Bi_2_Br_9_ thin films via CVD for X-ray detection applications.^67^ Using a powder-based CVD process with mixed CsBr and BiBr_3_ precursors, pinhole-free films with compact grain structure and high crystallinity were obtained by tuning the deposition temperature and growth duration. As shown in the SEM images in Figure 3D, varying the CsBr:BiBr_3_ molar ratio has a profound effect on film morphology. At a lower molar ratio of CsBr:BiBr_3_, or BiBr_3_-rich conditions, films are comprised of loosely distributed submicron (<1 μm) spherical grains, which can hinder carrier transport and degrade device performance. At a higher CsBr:BiBr_3_ ratio, the films display uniform grain size. The resulting devices exhibit exceptional performance metrics with an ultralow dark current density of 0.47 nA cm^–2^, highlighting their potential for sensitive radiation detection.

Another key advantage of CVD is the ability to achieve epitaxial growth, which is challenging to realize in solution processes. For example, CVD-grown epitaxial CsPbBr_3_ thin films on SrTiO_3_ (100) substrates exhibited high crystallinity, narrow rocking curve widths (full width half maxima, FWHM = 0.18°), low defect densities (∼10^12^ cm^–3^), and electron mobilities exceeding 500 cm^2^/V·s, comparable to bulk single crystals.^96^ This study illustrates how epitaxial control not only improves film quality but also enables heteroepitaxy on lattice-mismatched substrates. Further advances in CVD epitaxy have demonstrated the potential of wafer-scale single-crystal perovskite films. These advances are reported by high-temperature CVD of CsPbBr_3_, CsSnBr_3_, and mixed Sn/Pb halide perovskites on alkali halide substrates, yielding mirror-flat, grain boundary-free thin films with long carrier lifetimes and high PL quantum yield (PLQY).^97^ More recently, graphene-coated polar substrates enabled the growth of epitaxial CsPbBr_3_ films with significantly reduced dislocation densities, enhanced crystallinity, and improved optoelectronic performance.^98^ These studies highlight the ability of CVD to create high-quality, epitaxial thin films with enhanced structural and electronic properties.

### 3.3 MOCVD-Grown Thin Films

Despite its industrial maturity as a technique widely used for III–V and other compound semiconductors, MOCVD has seen limited application to MHPs, primarily due to the lack of suitable volatile precursors. Traditional MHP precursors, typically low-vapor pressure solid halide salts, undergo sublimation and rapid condensation on substrates, which does not provide a sufficient level of independent vapor-phase control of *each* component required for MOCVD. However, a recent breakthrough study reported a successful MOCVD deposition of MHPs films using carefully chosen volatile, thermally stable precursors for each of the three primary components—metal, halide, and amine.^28^ In this study, tetraethyllead (PbEt_4_) is employed as the volatile lead source, along with hydrogen halide gases (HBr and HI) and methylamine (MA^0^) gas as halide and MA cation sources, respectively. The MOCVD system enables precise independent delivery of the organometallic precursors simultaneously under controlled conditions, as illustrated in the schematic of Figure 4A. Within this dual-zone system, a high-temperature zone (*T*_1_) activates the PbEt_4_ precursor, while a lower-temperature zone (*T*_2_) supports perovskite film formation on the substrate. Figure 4A also shows the top and side-view SEM images of the resulting films. In addition to this, a phase diagram shown in Figure 4B details the process window for forming phase-pure MAPbI_3_. This work marks a significant milestone by demonstrating the feasibility of MOCVD for MHP synthesis and highlights the potential of this method to provide near-atomic-level control over film stoichiometry and thickness, capabilities that have made MOCVD indispensable in III–V semiconductor manufacturing.

**Figure 4:**
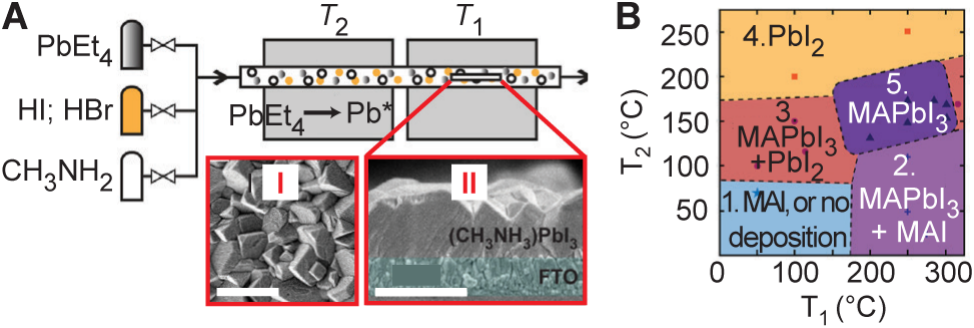
MOCVD processed thin films. (A) Schematic illustration of MOCVD for MAPbI_3_ thin films with SEM images of the top (I) and cross-sectional (II) view; scale bars: 1 μm. (B) Illustration of the phases deposited on substrates as a function of *T*_1_ and *T*_2_. Reproduced from ref. 28 Copyright 2025 American Chemical Society.

### 3.4 Thin Films Grown by Hybrid and Sequential Chemical Conversion

Recent advances in sequential vapor-phase growth techniques have enabled the fabrication of high-performance thin-film transistors. Notably, CsSnI_3_ thin film transistors have been realized through a multistep thermal evaporation method, where each component, PbX_2_, SnI_2_, and CsI, is sequentially deposited onto substrate (Figure 5A).^85^ PbX_2_ is deposited as the initial layer, followed by SnI_2_ and CsI layers. The multilayer films are subsequently annealed to form a CsSnI_3_:PbX_2_ film. PbX_2_ serves as a solid-state reaction initiator during the vacuum coevaporation process, enhancing the conversion of the precursor components into the desired high-quality perovskite phase. Devices fabricated using CsSnI_3_:PbCl_2_ films demonstrated remarkable field-effect hole mobilities exceeding 30 cm^2^/V·s, on/off ratios around 10^8^, high reproducibility, and improved ambient stability. Beyond Pb and Sn, sequential vapor deposition has also been employed for DP, exemplified by the growth of Cs_2_AgBiBr_6_ thin films with high phase purity and stability.^100^

**Figure 5:**
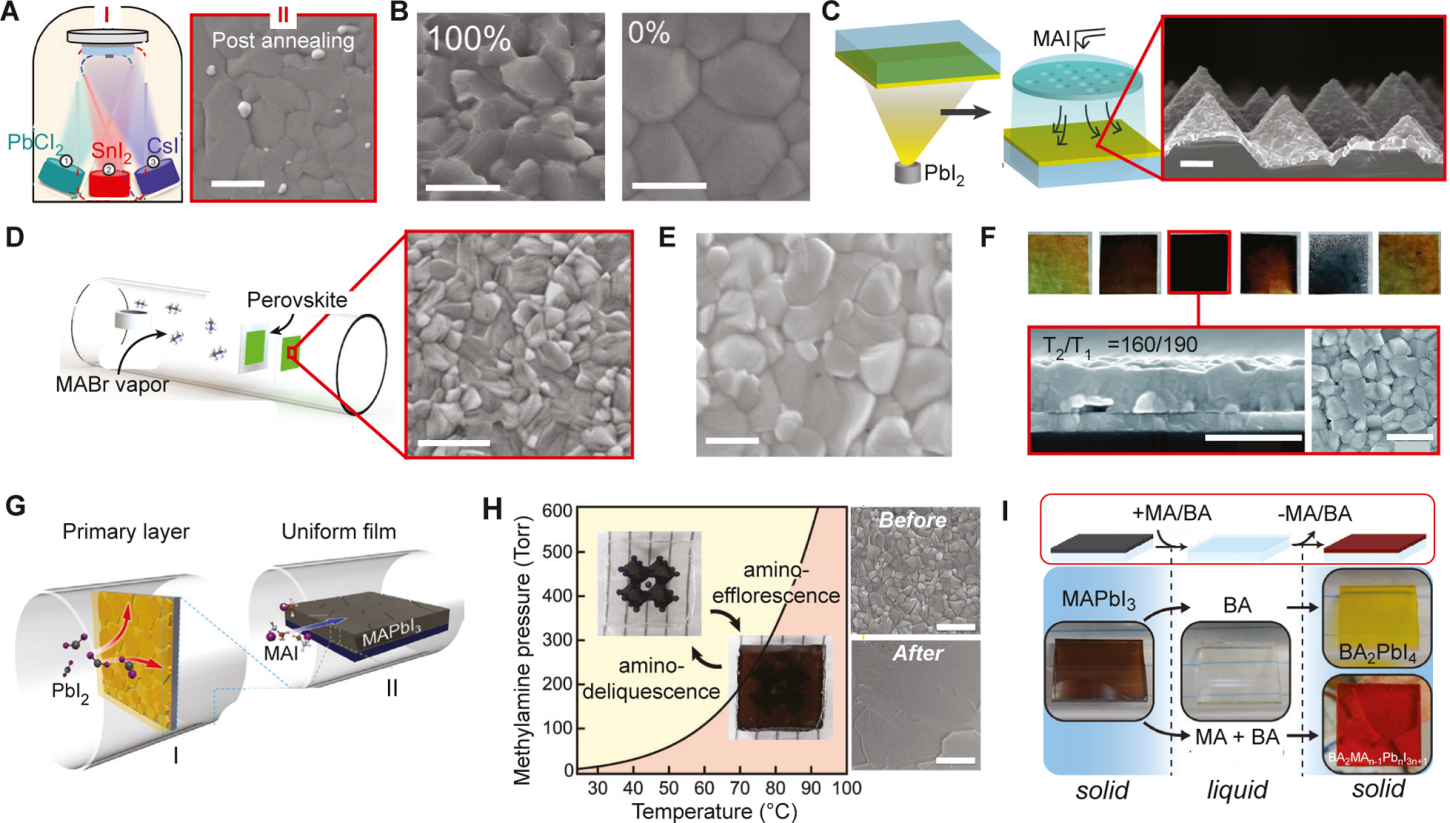
Thin films prepared by hybrid/sequential chemical conversion. (A) Sequential evaporation of precursors (I), and SEM images of CsSnI_3_:PbI_2_ film (II); scale bar: 2 μm. (B) SEM images of films with varying BAI-to-MAI ratios, 100% BAI (left) and 0% BAI (right); scale bars: 500 nm. (C) Schematic of the VTD (left) and SEM image (right) of MAPbI_3_ film; scale bar: 2 μm. (D) Schematic of reaction chamber (left) and SEM image of MAPbBr_3_ film (right); scale bar: 2 μm. (E) SEM image of the MAPbI_3_ film; scale bar: 500 nm. (F) Optical images at different *T*_2_/*T*_1_ (top) and SEM images at *T*_2_:160/*T*_1_:190 conditions (bottom); scale bars: 2 μm. (G) Schematic of PbI_2_ (I) to MAPbI_3_ film (II) conversion. (H) Phase diagram with SEM images of before and after amino efflorescence; scale bar: 500 nm (top) and 150 μm (bottom). (I) Conversion of MAPbI_3_ film in a mixed MA^0^ vapor environment. (A) Reproduced from ref. 85 Copyright 2021 American Chemical Society. (B) Reproduced with permission from ref. 99 Copyright 2021 Elsevier. (C) Reproduced from ref. 86 Copyright 2021 American Chemical Society. (D) Reproduced from ref. 87 Copyright 2017 American Chemical Society. (E) Reproduced from ref. 88 Copyright 2015 American Chemical Society. (F) Reproduced with the permission from ref. 89 Copyright 2020 Royal Society of Chemistry. (G) Reproduced from ref. 90 Copyright 2023 American Chemical Society. (H) Reproduced from ref. 91 Copyright 2021 American Chemical Society. (I) Reproduced from ref. 92 Copyright 2023 American Chemical Society.

Two step sequential PVD growth have also enabled investigations into the influence of various ammonium-based organic salts (R-NH_3_I) on the structural and electronic properties of perovskite films.^99^ In this study, a set of R-NH_3_I salts—including butylammonium iodide (BAI), phenylethylammonium iodide (PEAI), pentylmethylammonium iodide (PMAI), allylammonium iodide (ALI) and MAI—were sequentially deposited under high vacuum conditions following initial vapor-phase deposition of the inorganic framework. This approach facilitated precise control over the incorporation and distribution of organic cations within the perovskite lattice, allowing for tailored dimensionality (i.e., formation of 2D/3D layered heterostructures) and targeted defect passivation depending on the functional group’s electron-donating strength. These layered perovskite structures are represented by the general RP formula (R-NH_3_)_2_A_*n*__–1_B_*n*_X_3__*n*__+1_, where *n* denotes the number of inorganic layers separating adjacent organic cations. As shown in Figure 5B, systematic variation of the BAI-to-MAI ratio induced pronounced changes in grain morphology. Among the organic cations studied, devices incorporating ALI exhibited superior performance, achieving PCEs of 18.23% for small-area (0.045 cm^2^), and 15.48% for large-area (1 cm^2^) solar cells, along with significantly improved operational stability. Similar approach has been employed to grow MHPs, where MAI vapor successfully converts a predeposited PbI_2_ film into MAPbI_3_ using a custom-built vapor transport deposition (VTD) system equipped with a showerhead for MAI vapor as depicted in Figure 5C (left).^86^ This approach uniquely enables deposition over nonplanar, textured silicon substrates (Figure 5C, right) and demonstrated extended carrier diffusion lengths and suppressed recombination rates. Sequential growth approach has been also employed to produce MAPbBr_3_-based LEDs.^87^ This study begins with thermal evaporation of PbBr_2_ deposition on substrate, followed by CVD conversion using MABr vapor, as depicted in the schematic in Figure 5D (left). This approach yields films with exceptional surface smoothness (roughness <10 nm) and well-defined grain structures (Figure 5D, right), enabling LEDs with a maximum luminance of 560 cd/m^2^ and enhanced electroluminescent performance. Building on these sequential processes, a low-pressure (LP) CVD technique has been developed for the synthesis of planar-heterojunction perovskite solar cells.^88^ The two-step process involves an initial LPCVD deposition of PbI_2_, followed by the conversion to MAPbI_3_ perovskite through direct exposure to MAI vapor. Operating at low pressures slows the reaction kinetics, allowing a more controlled and complete conversion of PbI_2_ to MAPbI_3_. The resulting films are dense and pinhole-free, as shown in Figure 5E, and resulting solar cell devices fabricated using this technique exhibit a PCE of 12.7% under high humidity conditions (>60%).

Sequential growth methods have also contributed to kinetic studies of MHPs formation. For instance, mixed-cation perovskite films of nominal composition (Cs_0.04_FA_0.96_)­PbI_3_ were synthesized via a controlled two-step CVD process. A metal-halide precursor layer is first evaporated onto substrate, followed by exposure to FAI vapor under tightly regulated isothermal and isobaric conditions inside a specially designed tubular reactor. The key to this process is the independent control of the FAI source temperature (*T*_1_) and the substrate temperature (*T*_2_). Varying *T*_2_ revealed that lower substrate temperatures favored nucleation-limited growth with smaller grains, while higher temperatures led to diffusion-limited growth with larger grains, but increased risk of thermal decomposition or incomplete conversion. Images representing the deposition at different *T*_2_ */T*_1_ conditions are shown in Figure 5F, with an SEM image showing a sample synthesized at *T*_2_:160/*T*_1_:190. Using this optimized method, semitransparent planar solar cells yield a PCE of 9.6%. Substrate orientation has also been shown to play a critical role in film uniformity during sequential growth.^90^ A two-step deposition strategy was employed in which PbI_2_ was first deposited, followed by MAI vapor exposure (Figure 5G). Systematic analysis of substrate orientation revealed that a vertical orientation of the substrate during the PbI_2_ deposition results in a uniform film thickness and a horizontal orientation of the substrate during the MAI conversion step enhanced intercalation and nucleation, yielding films with greater uniformity and consistent grain morphology. The photoconductor arrays fabricated using the vertical to horizontal substrate orientation (vertical deposition of PbI_2_ followed by horizontal conversion with MAI) demonstrated significantly enhanced device performance compared to the horizontal to vertical configuration.

An innovative yet distinct approach in vapor-phase perovskite processing has introduced the phenomena of “amino-deliquescence” and “amino-efflorescence,” describing the dynamic transformation of MAPbI_3_ films under exposure of MA^0^ vapor.^91^ Notably, this process involves a neutral MA^0^ vapor rather than MA salts. *In-situ* extinction spectroscopy and optical microscopy allow mapping of a pressure–temperature phase diagram (Figure 5H), revealing a hysteresis loop between the liquefaction (amino-deliquescence) and recrystallization (amino-efflorescence), suggesting a nucleation barrier during the recrystallization process. During amino-deliquescence, the perovskite spontaneously and exothermically dissolves in MA^0^ vapors (Δ*H* = −96 kJ/mol), forming a condensed intermediate liquid at MA^0^ partial pressures far below equilibrium. Amino-efflorescence drives the reverse recrystallization process, producing highly crystalline, millimeter-scale grains with dramatically reduced defect densities, as shown in the SEM images in Figure 5H. Extending this framework, amino-mediated vapor processing has been applied to compositionally tune quasi-2D RP hybrid perovskites (BA)_2_(MA)_*n*‑1_Pb_*n*_I_3__*n*+1_.^92^ Exchange of amines occurs in the liquid amino-deliquesced state, causing a different film stoichiometry and phase to appear after amino-efflorescence, as illustrated in Figure 5I. By carefully controlling the partial pressures of mixed MA^0^ and BA^0^ vapors during amino-deliquescence/efflorescence cycles, a linear relationship is established between vapor composition and film stoichiometry, following Raoult’s law. This enables tunable incorporation of multiple cations that directly controls layered dimensionality, bandgap, and optoelectronic characteristics.

## 4 MHP Nanostructures

Vapor-phase techniques provide new opportunities for the controlled growth of single-crystalline micro- and nanostructures with a range of shapes and sizes. In this section, we summarize notable advances in the vapor-phase synthesis of 1D NWs (section 4.1) (Table 5), 2D NPLs (section 4.2) (Table 6) and periodic arrays of microstructures (section 4.3) (Table 7).

**Table 5:** Summary of Vapor-Grown NWs

Material	Key Observations	Properties	Refs
MAPbX_3_	Conversion of single-crystalline PbX_2_ NWs to MAPbX_3_	Demonstrates anisotropic layer-by-layer growth along *c*-axis; template for subsequent halide-conversion processes	^101^
MAPbI_3_	Complete vapor-phase MAI conversion preserves NW morphology; smooth surfaces; VLS	Direct conversion enables single-crystal MAPbI_3_ NWs with efficient photoconductivity and PL response	^24^
CsPbBr_3_	One-step VLS growth produces compact, monodisperse CsPbBr_3_ NWs with micrometer-scale length	Exhibits strong excitonic emission; uniform halide incorporation and high crystallinity	^34^
CsPbX_3_	Oriented NW arrays grown epitaxially; alignment tuned by substrate plane	Demonstrates substrate-induced epitaxial confinement; enables array-based photonic and lasing applications	^102,103^
BA_2_PbBr_4_	Faceted 1D NWs grown using layered perovskite for first time	Morphological control, NWs formation within intermediate-temperature window	^35^

**Table 6:** Summary of Vapor-Grown NPLs

Material	Key Observations	Properties	Refs
CsPbBr_3_	One-step vapor-phase synthesis yields micrometer-scale NPLs with atomically flat terraces	Exhibits uniform thickness (∼100 nm) and bright green PL with narrow FWHM	^106^
MAPbBr_3_	Low pressure → large flakes; optimal pressure + temperature → smooth single-crystal NPLs	Demonstrates pressure-temperature control of lateral size and crystallinity; uniform optical emission	^33^
MAPbI_3_	Two step → layered PbI_2_ to MAPbI_3_ NPLs; AFM confirms atomic-layer steps	Provides high-quality 2D precursors for conversion to MAPbI_3_; reveals substrate-controlled vdW growth	^107^
MAPbI_3_	Two-step vapor intercalation at 380–400 °C preserves NPL morphology	Enables lateral-size tuning and lasing (WGM cavity, threshold ≈ 17 μJ cm^– 2^)	^30^
Ag-doped Cs_3_Bi_2_Br_9_	Interlayer doping in vacancy-ordered 2D perovskites	Ag^+^ intercalation modifies electronic structure, enhances PL intensity; excitonic emission via bound-exciton formation	^29^
Mn^2+^-doped (BA)_2_PbBr_4_	Mn^2+^ substitution with lattice contraction (∼9.7%) and in-plane shear (∼6°) for first time	Correlation between dopant concentration and induced distortion; paramagnetic response	^108^

**Table 7:** Summary of Vapor-Processed Micropatterns

Material	Key Observations	Properties	Refs
MAPbI_3_	Photolithographically defined template confines PbI_2_ deposition; subsequent MAI vapor conversion yields patterns	Enables direct patterned growth of perovskite microarrays without post etching; compatible with flexible substrates	^70^
MAPbI_3_	Lithographically defined patterns filled by amino-deliquesced MAPbI_3_	Single-crystal microstructures; photodetectors; high responsivity (0.58 A W^–1^), and specific detectivity ≈ 4.8 × 10^13^ Jones	^110^

### 4.1 Micro/Nanowires

Adapting two-step vapor-phase processes, single-crystalline NWs of hybrid lead halide perovskites, including MAPbI_3_, MAPbBr_3_, and mixed-halide MAPbI_*x*_Cl_3‑x_ have been successfully synthesized.^101^ In the first step, PbX_2_ NWs are grown directly on substrate via catalyst-free CVD. These precursor NWs are subsequently converted *in situ* to perovskite NWs through exposure to MAX vapor at approximately 150 °C, facilitating a complete solid–vapor reaction that preserves the original NW morphology, as shown by SEM images in Figure 6A. The synthesized NWs exhibit strong optical confinement and remarkably low lasing thresholds (∼220 nJ/cm^2^), enabling tunable room-temperature nanolasers with emission wavelengths spanning 530 to 790 nm, depending on halide composition.^101^

**Figure 6:**
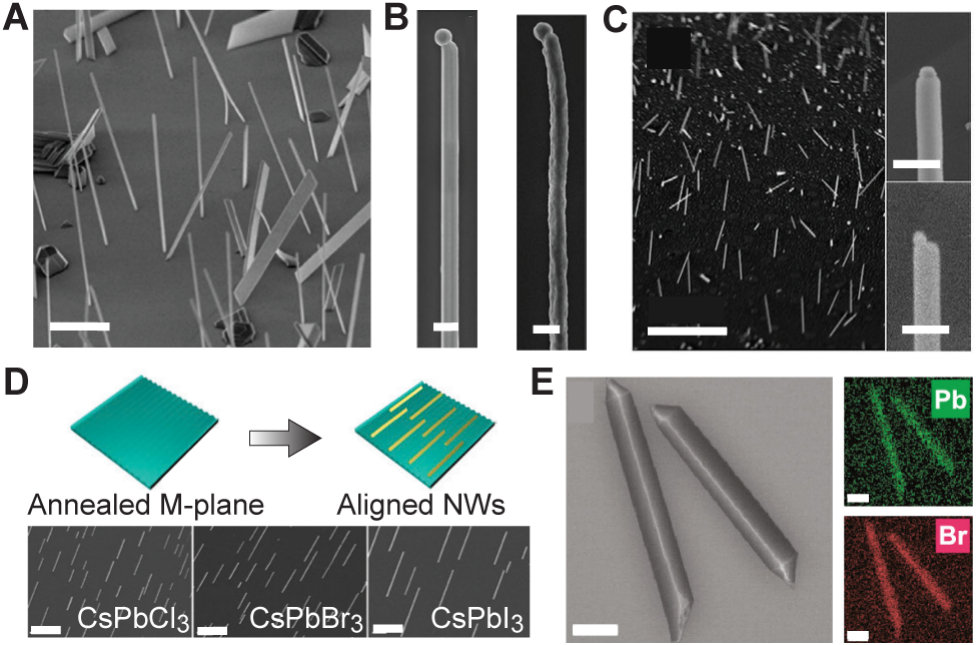
NWs growth. (A) SEM image of PbI_2_ NWs; scale bar: 5 μm. (B) SEM images of PbI_2_ NWs (left) and MAPbI_3_ NW (right); scale bars: 1 μm. (C) SEM images of CsPbBr_3_ NWs; scale bars: 30 μm (left), 200 nm (both insets). (D) Schematic of CsPbX_3_ NWs grown on an annealed M-plane sapphire (top) and SEM images of the aligned CsPbCl_3_, CsPbBr_3_, and CsPbI_3_ NWs; scale bars: 5 μm. (E) SEM images and elemental maps for Pb (green) and Br (red) of BA_2_PbBr_4_ NWs; scale bars: 2 μm. (A) Reproduced from ref. 101 Copyright 2015 American Chemical Society. (B) Reproduced from ref. 24 Copyright 2017 American Chemical Society. (C) Reproduced from ref. 34 Copyright 2019 American Chemical Society. (D) Reproduced from ref. 102 Copyright 2018 American Chemical Society. (E) Reprinted with permission under a Creative Commons CC-BY 4.0 from ref. 35 Copyright 2023 American Chemical Society.

A recent advancement is the novel self-catalyzed VLS growth of single-crystalline PbX_2_ NWs (Figure 6B, left), followed by their conversion to MAPbI_3_ (Figure 6B, right).^24^ In this method, vapor-transported lead and halide precursors condense on the substrate to form liquid Pb^2+^ droplets on the substrate, which subsequently act as self-catalysts. These droplets absorb PbI_2_ vapor, become supersaturated, and drive the unidirectional growth of PbI_2_ NWs without the need for foreign metal catalysts. Subsequent low-temperature exposure to MAI vapor converts the PbI_2_ into MAPbI_3_, while retaining the high aspect ratio. The resulting NWs exhibit strong absorption anisotropy and an enhanced optical antenna effect. Direct, one-step VLS growth of perovskite NWs has also been demonstrated with all-inorganic CsPbX_3_.^34^ Here, Sn nanoparticles deposited on the substrate serve as catalysts. A two-zone tube furnace is employed, where a source powder mixture composed of CsX and PbX_2_ is vaporized in the high-temperature zone (430–470 °C). The vaporized precursors are then transported by an Ar/H_2_ (80:15) gas mixture to the low-temperature zone (290–330 °C), where they interact with the Sn catalysts to initiate CsPbX_3_ NW growth at the liquid–solid interface. This process yields vertically aligned NW arrays with uniform diameters (∼150 nm) and single-crystallinity, as shown in Figure 6C. Photodetectors fabricated from individual NWs demonstrate high photoresponsivity exceeding 4489 A/W and detectivity over 7.9 × 10^12^ Jones in the visible light spectrum. Additionally, field-effect transistors (FETs) based on these NWs achieve superior hole mobility up to 3.05 cm^2^ V^–1^ s^–1^, surpassing the performance of previously reported all-inorganic lead halide perovskite devices. Precise spatial control represents the pinnacle of NWs synthesis advancement.

Early demonstrations of vapor-phase NW growth showed epitaxial CsPbX_3_ NW arrays synthesis on mica substrates. The growth produced triangular prism-shaped NWs with lengths from tens of microns to millimeters, with the cubic phase of CsPbI_3_ stabilized at room temperature.^103^ In a complementary approach, shown in Figure 6D, vapor-phase templated growth produces aligned, all-inorganic CsPbX_3_ NWs arrays directly on annealed M-plane sapphire substrates.^69,102^ By sublimating CsX and PbX_2_ powders in a controlled high-temperature zone, centimeter-scale, in-plane alignment with uniform diameters (∼100–300 nm) and high crystallinity are synthesized (Figure 6D, bottom). These NWs demonstrate room-temperature lasing with pumping thresholds as low as ∼10 μJ/cm,^2^ high-quality factors (*Q* > 1000), and strong, composition-dependent Rabi splitting energies, indicating robust exciton-photon coupling.

Growing 1D NWs with 2D perovskites is challenging due to their layered lattice structures and strong tendency to form sheet-like morphologies, unless specific chemical binding strategies are introduced to template their growth.^104,105^ A recent study has shown that controlled CVD conditions can predominantly yield NWs of the layered *n* = 1 BA_2_PbBr_4_ by balancing the growth rates along orthogonal directions.^35^ In this process, PbBr_2_ and BABr precursors are placed at separate upstream furnace zones under Ar flow, allowing for controlled codelivery of vapors to the substrate. Systematic exploration of temperature and pressure conditions revealed that crystal morphology is strongly temperature dependent. At high temperatures, thin nanosheets are favored; at intermediate temperatures, growth proceeds via the initial formation of 3D pyramidal nuclei that elongate into NWs upon continued deposition (Figure 6E). This behavior is attributed to temperature-dependent diffusion of surface species. The resulting BA_2_PbBr_4_ NWs exhibit strong excitonic emission and offers promising morphologies to form optical cavities in layered crystal structures with low lasing thresholds.

### 4.2 Micro/Nanoplatelets

NPLs with thicknesses below 100 nm are nanostructures well suited for integration into ultrathin optoelectronic devices. Van der Waals (vdW) epitaxy has emerged as an effective strategy for controlled crystal growth showcased in early studies for MAPbBr_3_ NPLs^109^ and with recent demonstrations of high-quality single-crystalline CsPbX_3_ NPLs on mica substrates.^106^ Using a 1:1 molar ratio of PbX_2_ and CsX as source materials, optimized growth conditions for all-inorganic CsPbCl_3_, CsPbBr_3_, and CsPbI_3_ were identified as 625 °C and 100 Torr, 575 °C and 50 Torr, and 550 °C and 100 Torr, respectively. The resulting well-aligned NPLs exhibit lateral dimensions between 1.0 and 20.0 μm, thicknesses ranging from 50 to 300 nm, as shown in Figure 7A, and remarkably smooth surfaces with a root-mean-square (rms) roughness of approximately 2.0 nm. Most remarkably, these NPLs function as whispering gallery mode (WGM) microcavities and support low-threshold lasing (∼2.0 μJ/cm^2^) with ultranarrow line widths (0.14–0.15 nm), among the narrowest reported for single-crystalline semiconductor microcavities in the visible region. A one-step vapor-phase method has also been employed to fabricate single-crystalline MAPbBr_3_ NPLs on mica.^33^ Controlled low-pressure conditions ensure uniform vapor transport and condensation, and maintain optimal vacuum levels, providing steady precursor flux preventing unwanted nucleation and promoting large, single-crystalline NPL growth. Substrate temperatures between 120 and 150 °C yield smooth, well-ordered NPLs with enhanced crystallinity and minimized defects. This fine-tuned control enables precise manipulation of NPL size, thickness, (Figure 7B) and optical quality.

**Figure 7:**
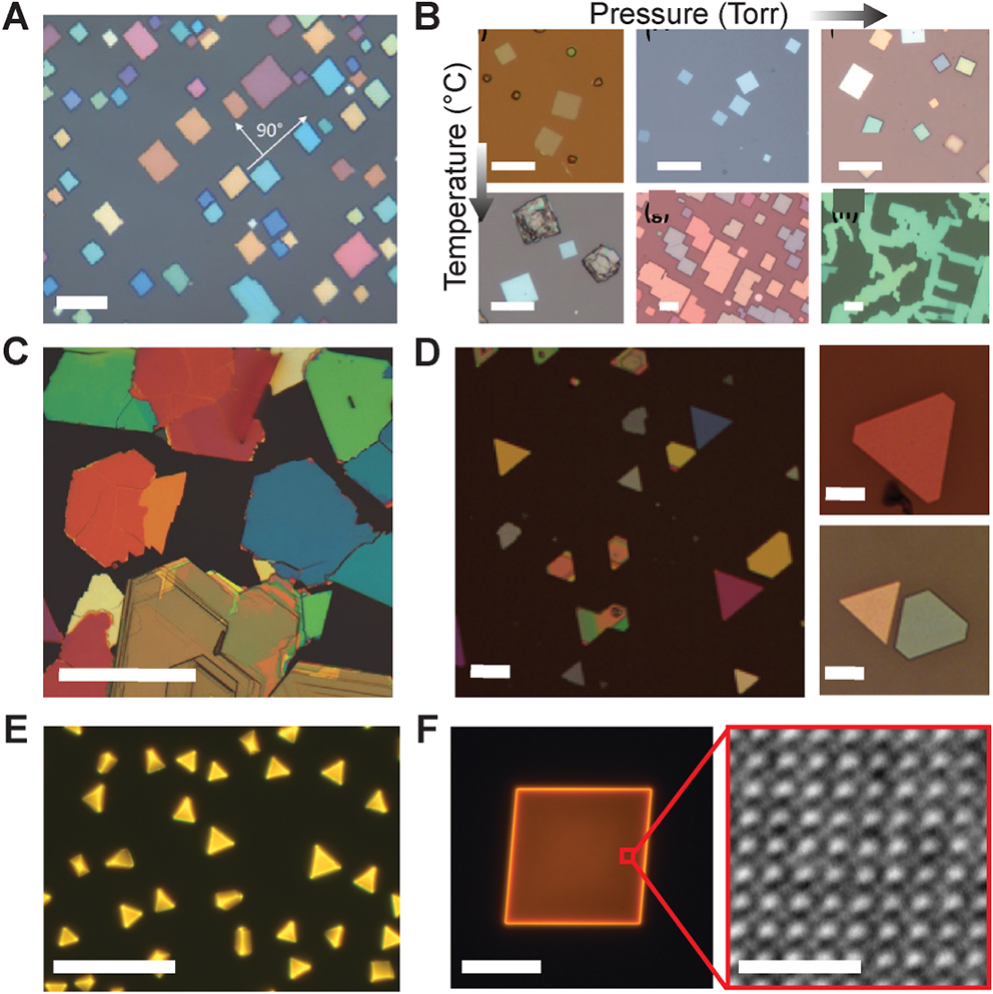
NPLs growth. Optical images of (A) CsPbBr_3_ NPLs; scale bar: 10 μm, (B) showing effects of temperature (top to bottom) and pressure (left to right) on the morphology of MAPbBr_3_ NPLs; scale bars: 10 μm, (C) PbI_2_; scale bar: 20 μm, (D) PbI_2_ NPLs (left), MAPbI_3_ NPLs at 380 °C (bottom right), and 400 °C (top right) reaction conditions; scale bars: 40 μm, (E) Ag-doped CBB; scale bar: 40 μm, (F) PL (left) and STEM (right) images of Mn^2+^-doped (BA)_2_PbBr_4_ NPL; scale bars: 20 μm (left), 1 nm (right). (A) Reproduced with the permission from ref. 106 Copyright 2016 John Wiley and Sons. (B) Reproduced from ref. 33 Copyright 2019 American Chemical Society. (C) Reproduced with the permission from ref. 107 Copyright 2014 John Wiley and Sons. (D) Reproduced with the permission from ref. 30 Copyright 2020 Elsevier. (E) Reprinted with permission under a Creative Commons CC-BY 4.0 from ref. 29 Copyright 2025 American Chemical Society. (F) Reprinted with permission under a Creative Commons CC-BY 4.0 from ref. 108 Copyright 2025 American Chemical Society.

VdW epitaxy has been adopted for organic–inorganic hybrid perovskite NPLs involving two steps reaction: growing PbI_2_ NPLs directly onto mica (Figure 7C), followed by thermal intercalation of MAI vapor, which transforms PbI_2_ into MAPbI_3_ while preserving the platelet morphology.^107^ These NPLs, with lateral sizes up to tens of micrometers, display different shapes and atomically smooth surfaces, achieve single-crystal quality, and exhibit exceptional optical properties, including PL with narrow line widths and electron diffusion lengths exceeding 200 nm, making them promising candidates for photovoltaic applications, where long diffusion lengths, enhanced charge transport, and optical coherence are critical. A similar approach has been adopted to grow smooth PbI_2_ NPLs (Figure 7D) first on mica, which were subsequently converted into MAPbI_3_ by exposure to MAI vapor.^30^ This study shows higher temperatures and longer deposition times facilitate lateral growth, while substrate positioning affects local vapor flux, all contributing to precise size modulation. These NPLs exhibit exceptional crystallinity and naturally form WGM microcavities, demonstrating lasing with a low threshold of ∼17 μJ/cm^2^.

In addition to morphological control, CVD methods have enabled novel doping strategies in layered perovskites to engineer light emission. For example, interlayer doping of vacancy-ordered 2D Cs_3_Bi_2_Br_9_ with Ag^+^ intercalation has been achieved by CVD growth.^29^ The synthesis involves introducing Ag^+^ atoms into the interlayer gaps of the Cs_3_Bi_2_Br_9_ lattice, effectively modifying its electronic structure through a controlled chemical reaction. Ag^+^ ions kinetically occupy neutral interlayer vacancy sites between the perovskite layers, ensuring uniform distribution without disrupting the host lattice. The optical image in Figure 7E shows significant enhancement of room-temperature PL emission, attributed to the formation of bound interlayer excitons at the Ag^+^ interlayer dopant sites.

Furthermore, an unusually large influence of dopant incorporation on the crystal lattice has recently been observed in CVD-grown BA_2_PbBr_4_ single crystals. Prior reports on Mn^2+^-doped semiconductor nanocrystals have largely resulted in surface doping and nonuniform distributions, showing minimal structural changes. Recent work demonstrates the successful growth of defect-free Mn^2+^-doped (BA)_2_PbBr_4_ single crystals using CVD, which enabled segregation-free uniform doping with significant lattice distortions—nearly an order of magnitude greater than previously reported.^108^ As a result, the square NPL crystal morphology of undoped BA_2_PbBr_4_ significantly distorts to a parallelogram NPL with in-plane angular distortion up to 6°, along with characteristic Mn^2+^ emission as shown in Figure 7F. The atom-resolved scanning transmission electron microscope (STEM) imaging clearly shows that the crystal distortion indeed originates from the atomic level strain. These findings with magnetic impurities of Mn^2+^ present a promising avenue for potentially achieving an even higher bulk incorporation of Mn^2+^ in large-area thin films with long-range magnetic ordering in ambient conditions for potential applications in spintronics and spin-photonics.

### 4.3 Array Patterning

Lithographic patterning of ordered arrays presents significant challenges due to the poor processability and stability of MHPs in common solvents during conventional lithographic patterning processes. The development of vapor-phase patterning techniques represents a critical advancement to overcome these limitations, enabling the formation of high-quality perovskite patterns with controlled morphology and reduced grain boundary density, which is essential for optimal device performance. These challenges have been successfully overcome by sequential CVD growth using lithographically defined polymer templates.^70^ The fabrication process shown in Figure 8A begins with the creation of photopatterned cross-linked copolymer templates on Si/SiO_2_ substrates. PbI_2_ is then selectively deposited *via* CVD, preferentially nucleating as well-aligned [0001]-oriented NPLs on exposed Si/SiO_2_ regions, while polymer-covered areas suppress nucleation and facilitate diffusion-limited growth, ensuring sharply defined patterns. These PbI_2_ structures are subsequently converted into MAPbI_3_ *via* a vapor–solid intercalation process using MAI vapor, preserving the integrity and geometry of the original patterns. The resulting perovskite arrays display smooth morphology and good crystallinity, as shown in Figure 8B.

**Figure 8:**
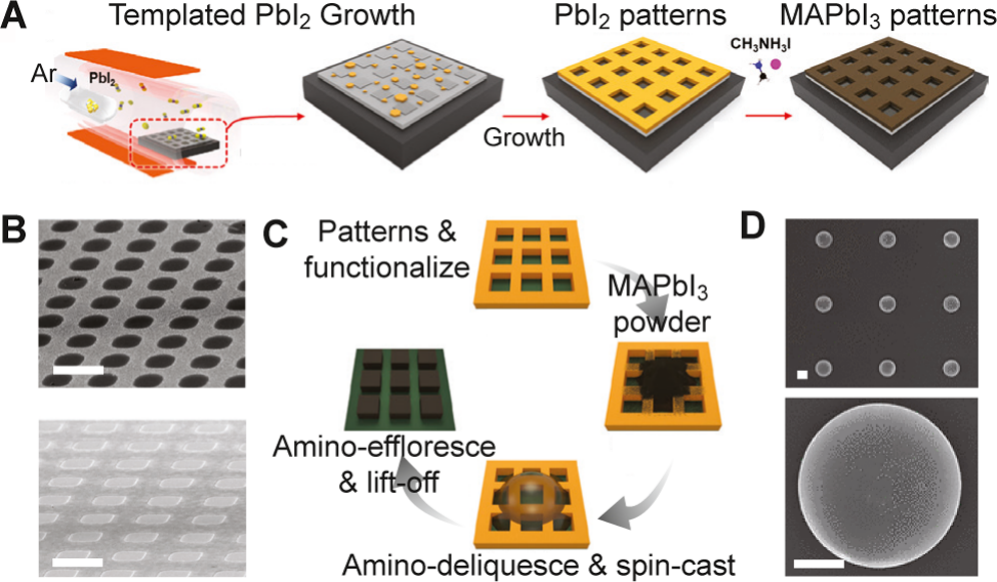
Micropatterns growth. (A) Schematic of PbI_2_ patterns on a template converted to MAPbI_3_. (B) SEM image of PbI_2_ thin films (top), and MAPbI_3_ films (bottom); scale bars: 50 μm. (C) Schematic of patterning process. (D) SEM images of MAPbI_3_ microstructures; scale bars: 1 μm. (A, B) Reproduced from ref. 70 Copyright 2019 American Chemical Society. (C, D) Reproduced ref. 110 Copyright 2021 American Chemical Society.

An innovative hybrid top-down/bottom-up approach has been developed to leverage amino-deliquescence to fabricate periodic MAPbI_3_ microstructures without the use of solvents.^110^ Figure 8C represents the stepwise methodology, starting with photolithography and chemical surface functionalization, which is employed to pattern substrates with distinct wetting properties. Solid MAPbI_3_ powder is placed on top of the patterns and then exposed to MA^0^ vapor, triggering amino-deliquescence, which liquefies the solid material. This liquified material is guided to selectively fill the patterned template areas while avoiding the regions that repel it. Upon removal of the MA^0^ vapor (amino-efflorescence), the perovskite recrystallizes within the defined regions. By carefully controlling nucleation and growth, the resulting crystal grains become significantly larger than the template features, resulting in true single crystals. Surface wettability quantified by contact angle measurements on various surfaces can be tuned to accommodate different lithographic resists and substrates. The microstructures (Figure 8D) are formed free from solvents and demonstrate excellent integration potential for optoelectronic devices.

## 5 Conclusion and Outlook

In summary, the recent advances in vapor-phase synthesis of MHPs open a broad design space for producing high-quality thin films, nanostructures, and array patterns with properties well-suited for various optoelectronic applications. The adoption of PVD, CVD, MOCVD, and hybrid methods enables tuning of interfaces, crystal orientation, and defect management, which are critical for optimizing device performance. Vapor-phase approaches have demonstrated exceptional potential in producing MHPs with superior phase purity, improved optoelectronic properties, and compatibility with unconventional doping, lithographic patterning, and integration into complex device architectures.

There remains room for further advancement in adapting other common vapor methods. For instance, atomic layer deposition (ALD), hybrid physical-chemical vapor deposition (HPCVD), and vapor-phase ion exchange methods have not yet been optimized for MHPs. ALD has revolutionized thin-film uniformity and interface control in III–V semiconductors but remains underexplored for metal halide precursors due to the complex chemistry of organic cations and the volatility of halides. Early demonstrations of ALD PbI_2_ films that can be converted into perovskite layers show great promise for conformal coating on textured or flexible substrates, potentially overcoming grain-boundary and pinhole-related stability issues.^111^ Similarly, HPCVD, which is widely used in high-quality epitaxial growth of superconductors and semiconductors like GaAs, could enable simultaneous vapor delivery and *in-situ* reaction of organic and inorganic precursors, improving crystal quality and doping control in MHP films.

Furthermore, several challenges remain for industrial adoption of vapor deposition of MHPs. Scalability is a key issue: while lab-scale devices routinely achieve uniformity and high performance, translating these methods to large-area modules requires an entirely new set of control in flux distribution and substrate handling.^112^ Process costs and yield are also critical factors; although vacuum systems enable reproducibility, they are more capital- and energy-intensive than solution processing, and long-term competitiveness will depend on whether the gains in film quality can offset these costs through higher device yield.^113^ Also, a performance gap still exists between solution-processed and vapor-deposited devices, particularly at record efficiencies, and further optimization of carrier lifetimes and interface quality will be essential to close it.^114^ From a film-property perspective, trap concentration, surface passivation, grain size, and carrier mobility remain bottlenecks that limit optoelectronic quality. Therefore, addressing these challenges with innovative strategies to implement additional vapor-phase techniques are expected, expanding the depth and breadth of MHP processing and offering a transformative pathway to scalable, high-performance, and stable perovskite optoelectronics.
